# Supplementary figures and images for: Flagellar Perturbations Activate Adhesion through Two Distinct Pathways in Caulobacter crescentus
Source: mBio. 2021 Feb 9;12(1):e03266-20. doi: 10.1128/mBio.03266-20 (PMC7885107; doi:10.1128/mBio.03266-20)

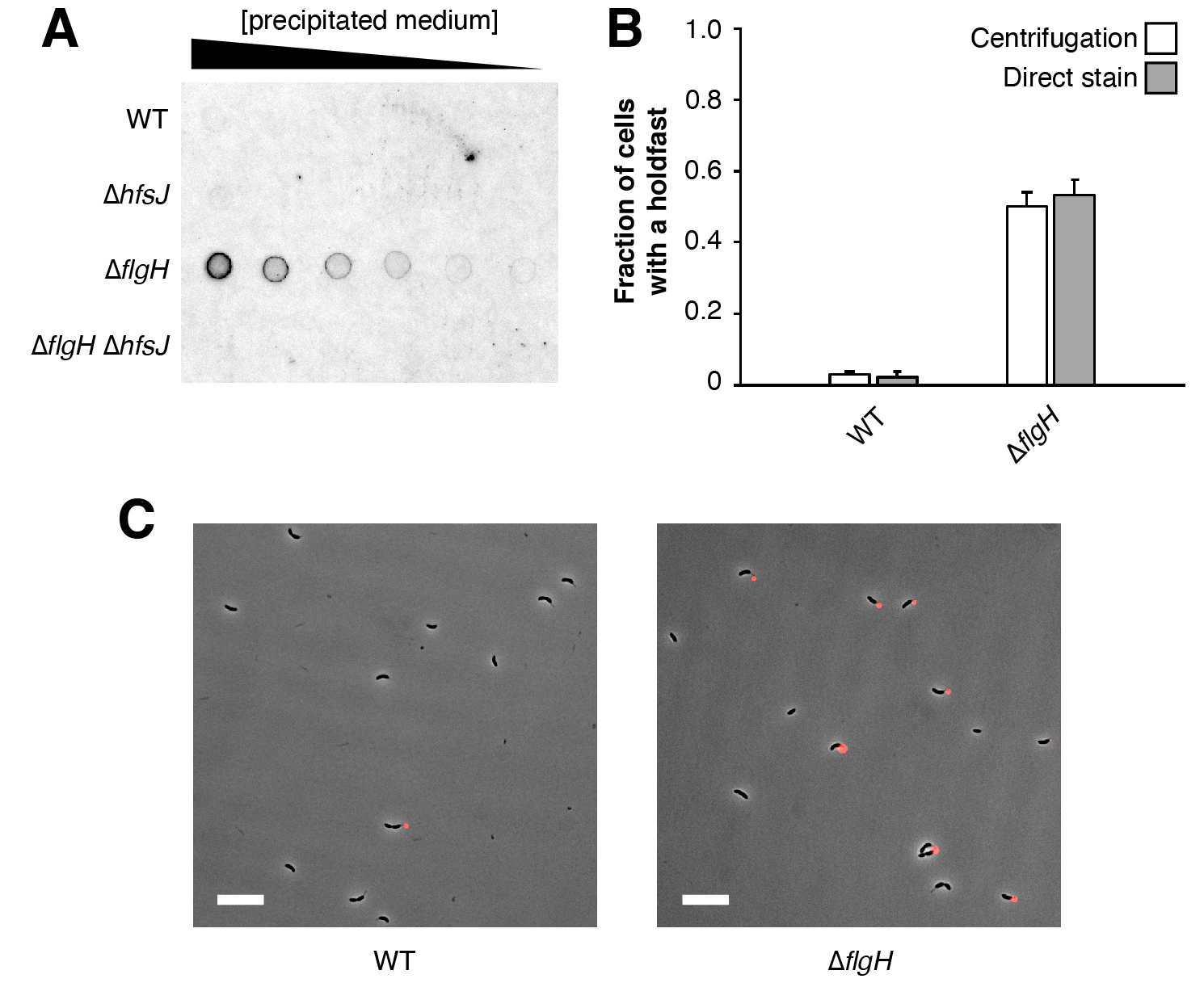

Supplement: FIG S1 [file mBio.03266-20-sf001.jpg]

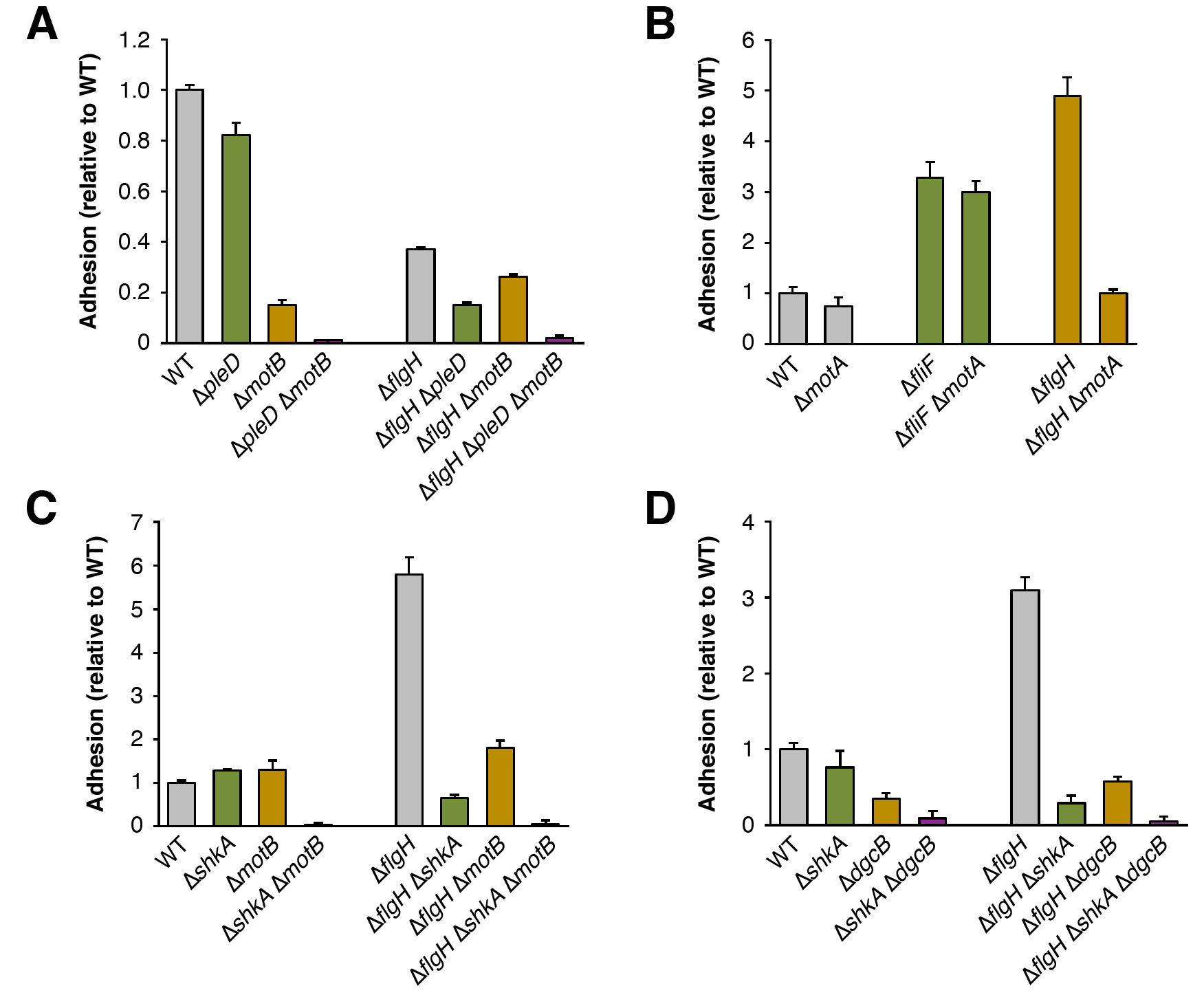

Supplement: FIG S2 [file mBio.03266-20-sf002.jpg]

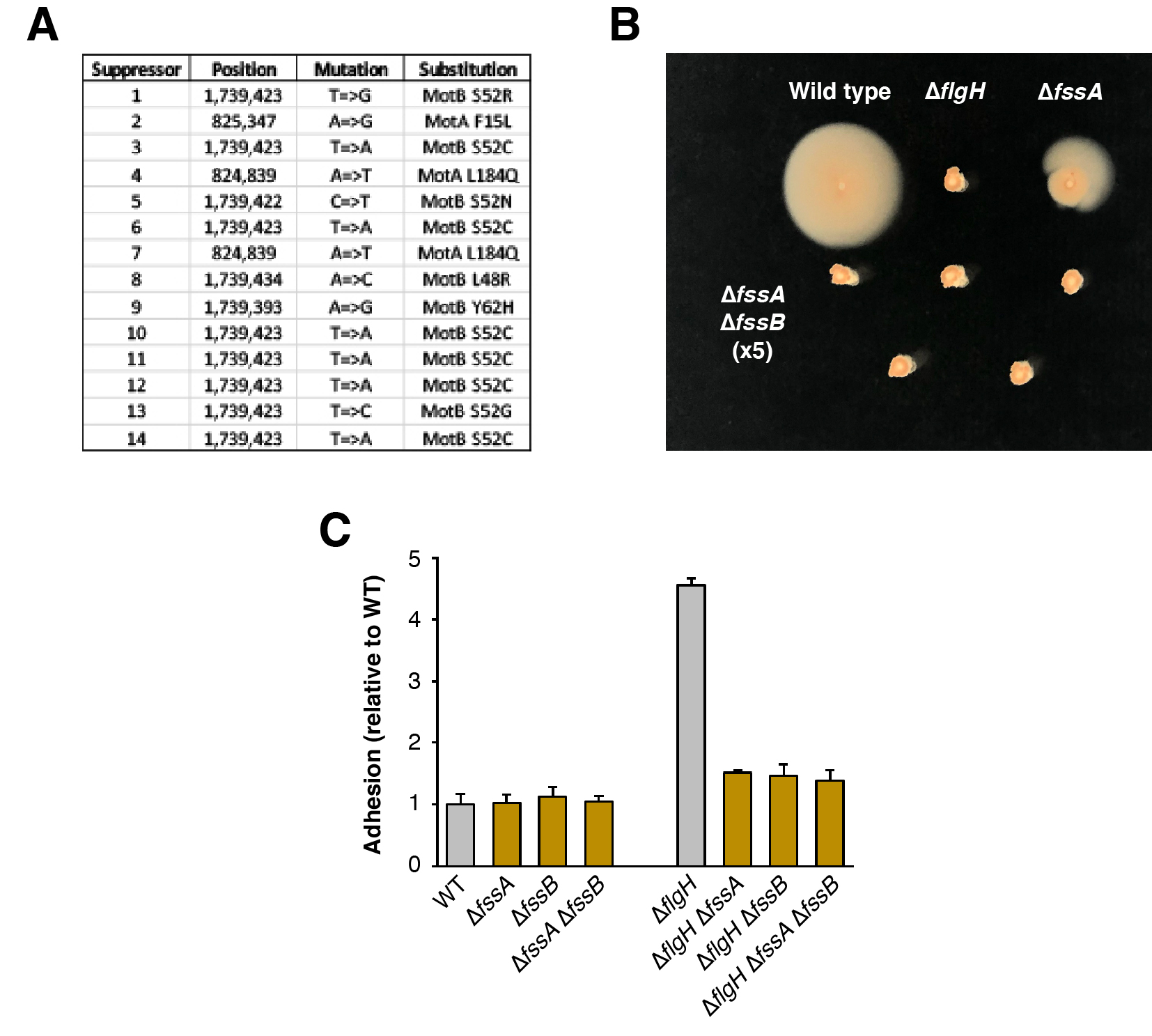

Supplement: FIG S3 [file mBio.03266-20-sf003.jpg]

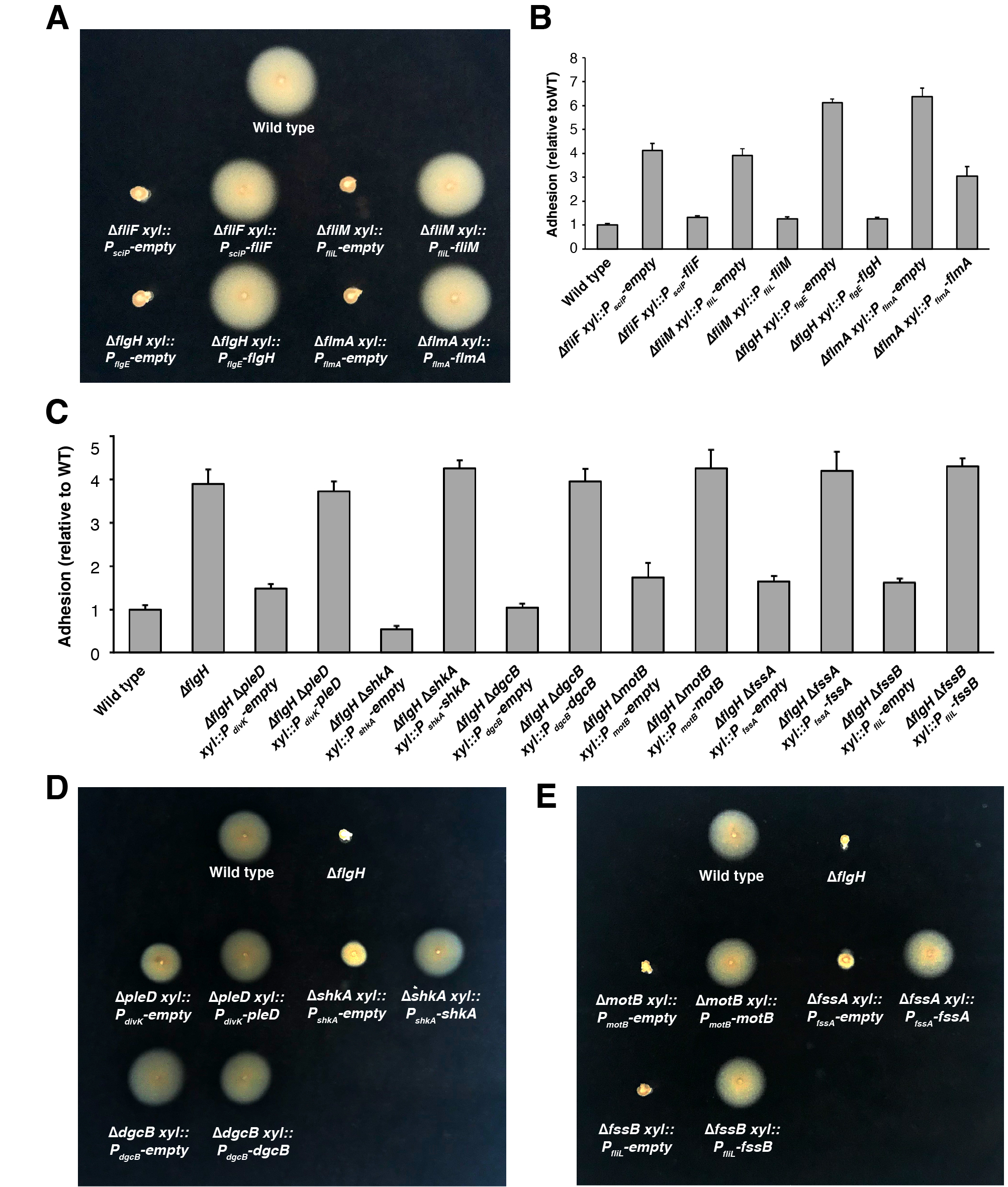

Supplement: FIG S4 [file mBio.03266-20-sf004.jpg]
